# Hysteresis and balance of backaction force on dielectric particles photothermally mediated by photonic nanojet

**DOI:** 10.1515/nanoph-2022-0312

**Published:** 2022-08-09

**Authors:** Yu-Xuan Ren, Gwinky G. K. Yip, Lei-Ming Zhou, Cheng-Wei Qiu, Jiawei Shi, Yi Zhou, Huade Mao, Kevin K. Tsia, Kenneth K. Y. Wong

**Affiliations:** Institute for Translational Brain Research, Shanghai Medical College, Fudan University, 200032, Shanghai, China; Department of Electrical and Electronic Engineering, University of Hong Kong, Pokfulam Road 999077, Hong Kong SAR, China; Department of Optical Engineering, School of Physics, Hefei University of Technology, 230601, Hefei, Anhui, China; Department of Electrical and Computer Engineering, National University of Singapore, 117583, Singapore, Singapore; Advanced Biomedical Instrumentation Centre, Hong Kong Science Park, Shatin, New Territories, 999077, Hong Kong, SAR, China

**Keywords:** cancer cell, counter-propagating beams, hysteresis, optical force, photonic nanojet, supercontinuum laser

## Abstract

Reversible control over the microparticle motion using light excites interesting applications in optofluidics, microswimmers, artificial optical matter, and biomedical engineering. The dielectric microspheres swim towards the near infrared pulsed laser in response to the backaction force mediated by photonic nanojet. Hereby, we report that the backaction force exhibits hysteretic behaviour owing to the distinguishable responses of the temperature rise inside the nanojet and the temperature rise of the liquid ensemble. Accordingly, the magnitude of backaction force at the same laser power varies between power increase and decrease stages. In order to develop multidimensional manipulation tool, we studied the possibility of using lasers with different spatiotemporal profiles to mediate the backaction force, and developed the counterpropagating beam scheme for reversible control of the particle motion directions. We further harness the hysteresis to reverse the direction of backaction force on dielectric particles in presence of a constant force from a counter-propagating beam with broadband supercontinuum spectrum. In contrast to the microsphere caught in the single beam gradient trap, the microsphere encounters augmented Brownian motion at higher balanced power level. The microsphere would eventually escape from the common region of the paired beams, enabling high throughput morphology analysis for cancer cell classification, biopsy, and diagnosis.

## 1 Introduction

Reconfigurable soft matter provides unique playground to investigate the fluid dynamics and the nonlinear physics under external stimuli. This includes the unconventional self-assembly of colloidal structures [1], and the underlying optomechanical interactions [2]. The control over the micro-/nano-swimmer is important for designing the targeted drug delivery system and nanosurgery, therefore multiple control schemes have been demonstrated using the ultrasound [3, 4], magnetic field [5, 6], optically generated hydrodynamic trap [7], and electric field [8] to self-propel the tiny swimmer in large scale. The laser light can be concentrated into diffraction-limited size to excite surface plasmon in the metal nanostructure, and can also control the motion of the biological micro-organism and the plasmonic nanomotor [9–12]. Since the discovery of the optical binding [13], and the concept of optical matter [1], the interplay of light and the particle suspension has attracted a great amount of interests. The spatially or temporally engineered laser could trigger the light–matter interaction and detects the nontrivial processes beneath the complex interplay, e.g., the nonlinear four wave mixing [14, 15], and self-trapping [16, 17]. The nonlinear bistability and hysteresis has been observed in single particle level in optical binding experiment [13, 18, 19]. During the past few decades, it has been well understood that the majority of those nonlinear effect has been associated with either optical gradient force or scattering mediated optical binding. Hence, it is unexplored to excite the nonlinear hysteresis through photothermal effect, which is of hitherto unsuspected importance, especially when the photothermal trapping flourishes in recent years [20–24].

Photons interact with the matter through momentum transfer, which brings about the forward radiation force on the absorptive particle [25]. The microparticle motion driven by the radiation pressure force follows the direction of photon momentum flux. However, the direction reversal of particle motion against the photon flux could be achieved through engineering of either the light wavefront or the optical refractive index of the background medium [26], and has also been realized in a plethora of experiments, e.g., the propagation-invariant beam, cylindrical vector polarization, and electromagnetic wave with plasmonic structures [27–30]. Single beam optical tweezers provide fine tool to exert force on and to measure the displacement of a single biomacromolecule, but face great challenges to increase the throughput. Parallelization using time-sharing or holography is capable of producing array of traps with high power requirement [31, 32], but is still insufficient for high throughput single-particle classification applications owing to the complexity in beam control and the increased power budget. For instance, the holographic array traps could manipulate multi-particles; however, the trapping positions have to be fine controlled to follow each particle. On application side, large-scale optical manipulation of micro-/nano-particles allows the creation of optical matter and the nanoscale heat engine [1, 33], [34], [35]. Although the photothermal effect has been applied for biomedical imaging [36] and photophoresis owing to the temperature gradient caused by inhomogeneous heating [37], the negative photophoretic force can also gather massive dielectric microparticles and to form cluster in a spindle-like region close to a fiber facet [38].

Since most probe for optical force is dielectric microparticle, and the microparticle itself is able to concentrate the light field into a subwavelength ‘jet’ with enhanced electric field energy, i.e., photonic nanojet (PNJ) [39, 40]. Although the PNJ is narrow, it offers optical gradient force for trapping of nanoparticles and bacteria [41], and mediate optogenetics [42]. However, the nanoparticle can only be manipulated when the particle is close to PNJ hindering the throughput of the nanoparticles. We have reported that the PNJ formed by concentrating a picosecond mode-locked laser pulse could mediate the backaction (antiparallel to the photon flux) on dielectric particles [43]. Our scheme is distinguishable from the conventional opto-thermoelectric microswimmer, which relies on the temperature gradient induced by two sides with different temperature responses to light (‘Janus particle’) [20]. Hereby, we identified interesting response of the backaction force on cancer cell to the change of laser power, i.e., the hysteresis; such nonlinear response provides reversible backaction force at the same power injection level. Further, we explore the use of continuous wave lasers with multiple laser lines and supercontinuum spectrum in the near-/mid-infrared region to pull the dielectric polymer particles and cancer cells. Moreover, we employ two laser beams shining the cancer cell suspension from opposite directions to provide balanced forces on the cells in a large scale of population. The balanced force would be produced under judicious control over the power ratio for the counter-propagating beams. The backaction force on cancer cells under counter-propagating beams may provide insights on how the cancer progression and treatment is affected by physical parameters and processes, i.e., the flow-induced stress, force altered micro-architecture, as well as change of the microenvironment. In general, the ability to manipulate cells with the counter-propagating beams suggests the potential application in high-throughput flow cytometry and cell sorting using the backaction force combined with the morphological profiling of biological cells. Finally, we demonstrate the use of the hysteresis to reverse the force direction in presence of the counterpropagating beams.

## 2 Results

### 2.1 Hysteresis on backaction force mediated by photonic nanojet

**Figure 1:**
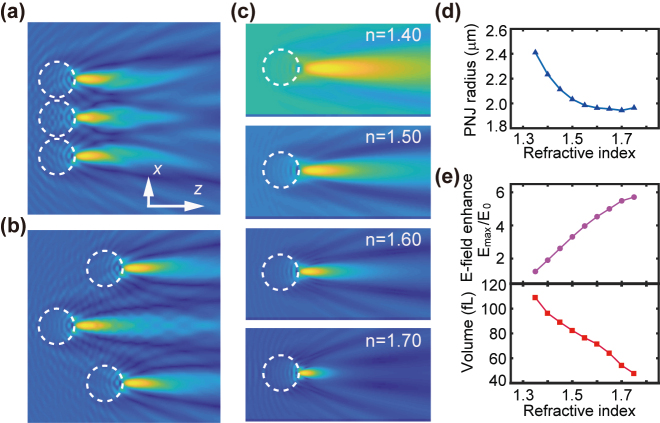
Self-induced PNJ innately moves along the microsphere. The PNJ formed by 2 μm-diameter polymer microspheres (dashed lines) aligned in (a) the same and (b) different transverse planes. (c) The sideview profiles of the light intensity formed with a 2 μm diameter polymer sphere with various values of optical refractive index. (d) The effective radius of the PNJ as function of the refractive index of the microsphere. (e) The maximum electric field enhancement inside the nanojet increases with the refractive index, while the effective volume of the PNJ decreases with the refractive index.

Optical tweezers use a high numerical aperture (NA) objective lens to concentrate light beam into a diffraction-limited spot. The microparticles would be attracted to the spot owing to the intensity gradient force. The trap region is close to the diffraction-limited focus which only occupies a volume on the order of femtoliter. For control over the particles far away from the trap center, the optical trap has to be mechanically scanned across a position close to the particle. Such inefficient optical manipulation fails to meet the applications in large-scale particle classification and biopsy, where parallel manipulation is required. The microsphere concentrates photons into PNJ with much augmented light energy, and passively moves towards the light source owing to the energy burst inside the nanojet [43]. Intuitively, we performed the finite-difference time-domain (FDTD) simulation to calculate the light field distribution in the shadow of microsphere. Figure 1(a) shows three dielectric polystyrene microspheres with 2 μm radius concentrate the light (illumination from left to right). The PNJ accompanies each microparticle with a constant distance determined by the sphere size and refractive index. No matter whether the microspheres are aligned in the same transverse plane (Figure 1(a)) or randomly distributed along the light propagation (Figure 1(b)), the PNJ always accompanies each microsphere with a fixed distance determined by the sphere size and relative refractive index. As a result, the microparticles would be ‘self-powered’ and move towards the light source individually without any geometric confinement in response merely to the PNJ mediated backaction force.

The PNJ formed by the 4 μm diameter microsphere with refractive indices of 1.4, 1.5, 1.6, and 1.7 are shown in Figure 1(c). The transverse size of PNJ decreases with the refractive index (Figure 1(c) and (d)). The electric field enhancement inside the PNJ, i.e., $e=\left|E_{\max}\right|/|E_{0}|$ , increases with refractive index (Figure 1(c) and (e)), here *E*_max_ is the maximum amplitude inside the nanojet, and *E*_0_ is the amplitude of the incident beam. Such enhanced electric field could even evaporate the liquid molecules inside the nanojet in combination with ultrashort femtosecond laser pulses [44]. The laser cavitation relies on the laser-induced breakdown to generate the air bubble to push the particle backwards. However, the ultrastrong electric filed could damage the microparticle such as the biological cells. In our previous report, we identified a photothermally mediated backaction force by the PNJ owing to the expansion of the liquid inside the nanojet. Assume the microsphere has a constant diameter immersed inside the medium with the same background refractive index; the PNJ has larger (smaller) volume for smaller (larger) refractive index of sphere (Figure 1(e)). This implies that the particle with refractive index close to but larger than the background would still be attracted by light. Therefore, biological cells, which have a weak refractive index contrast with the background medium, e.g., phosphate buffered saline (PBS), can also be pulled by light under the mechanism of PNJ.

The liquid molecules heat up inside the nanojet with a temperature rise from *T*_0_ to *T*. In response, the microsphere experiences a backaction force [43],

(1) $$F=\frac{V_{\text{jet}}\rho_{\text{sol}}}{2\tau }\sqrt{\frac{N_{A}k_{B}}{M_{\text{sol}}}}\left(\sqrt{T}-\sqrt{T_{0}}\right)$$

where *τ* is the particle response time (on the order of ∼ ms), *k*_B_ is Boltzmann constant, *N*_A_ is Avogadro constant, *M*_sol_ is the total number of solvent molecules within the nanojet volume *V*_jet_, and *ρ*_sol_ is the mass density of the solvent. The magnitude of force is much greater than the force produced by optical scattering, photophoresis, and thermophoresis.

**Figure 2:**
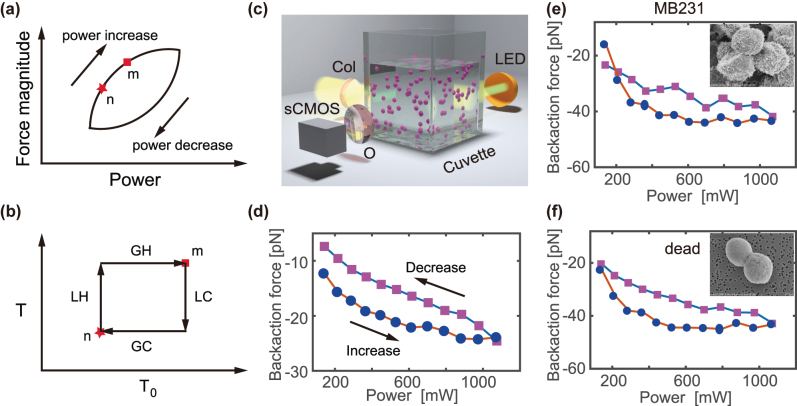
Force hysteresis on cancer cells. (a) Demonstration of backaction force hysteresis with respect to laser power. Markers show two representative states on the loading curve. (b) The back-action force was determined by four heating stages. LH, local heating; LC, local cooling; GH, global heating; GC, global cooling. *T*_0_ represents the ensemble temperature, and *T* denotes the temperature inside the nanojet. (c) Schematic of the experimental setup for the backaction force experiment. CMOS, complementary metal oxide semiconductor; LED, light-emitting diode; Col, collimator; O, objective. (d) The force hysteresis on the polystyrene microparticle with diameter of 5 μm. (e–f) The hysteresis for the cancer cells under different physiological conditions. The loading/unloading curve for (e) live and (f) fixed breast cancer cell (MDA-MB231). Insets show the SEM photos of MDA-MB231 cancer cells under (e) native and (f) ethanol immersion conditions. The magnitude of force is stronger in the power loading stage than in unloading stage with the same power when the hysteresis exists for both the standard polymer sphere and the cancer cell. Note that (a) shows the magnitude of force, while the “–” sign in (d–f) indicates the backward motion direction. Hence the force change direction is consistent between (a) and (d–f).

The magnitude of backaction force increases with the power injection monotonically as described in Eq. (1) and was corroborated by our previous experiment [43]. However, when the laser power further reduces, will the magnitude of backaction force reverse on the same path? Since the PNJ moves closely with the microsphere (Figure 1(b)), and the heat will diffuse once the microsphere moves away from the original position, thus, the background temperature would also increase homogeneously due to the heat diffusion. We consider two states for which the laser power increases from *p* ^(*n*)^ to *p* ^(*m*)^, the temperature for background and nanojet correspondingly increases from $T_{0}^{\left(n\right)}$ , *T* ^(*n*)^, to $T_{0}^{\left(m\right)}$ , *T* ^(*m*)^, with $T_{0}^{\left(n\right)}<T_{0}^{\left(m\right)}$ and *T* ^(*n*)^ < *T* ^(*m*)^ at steady state (Figure 2(a) and (b)). In equilibrium, the nominal force at each state would be,

(2) $$F^{\left(n\right)}=\sqrt{T^{\left(n\right)}}-\sqrt{T_{0}^{\left(n\right)}},$$

and,

(3) $$F^{\left(m\right)}=\sqrt{T^{\left(m\right)}}-\sqrt{T_{0}^{\left(m\right)}}.$$

Here, the constants in Eqs. (2) and (3) are the same as that in Eq. (1), and thus are omitted for simplicity. Note that *τ* is also assumed to be constant on the fact that the power for the two states is infinitely close to each other. Practically, the backaction temperature increases much slower than the temperature inside the nanojet, therefore the actual force with hysteresis approximates,

(4) $$F_{\text{hys}}^{\left(m\right)}\approx \sqrt{T^{\left(m\right)}}-\sqrt{T_{0}^{\left(n\right)}}.$$

The difference,

(5) $$F_{\text{hys}}^{\left(m\right)}-F^{\left(m\right)}\approx \sqrt{T_{0}^{\left(m\right)}}-\sqrt{T_{0}^{\left(n\right)}}>0$$

which implies that the backaction force increases faster than nominal force. The hysteretic force $F_{\text{hys}}^{\left(m\right)}$ is greater than the nominal value $F^{\left(m\right)}$ when the injected power ramps up. Similar analysis suggests a faster decrease of the force when the laser power is released from the system. The hysteretic backaction force is attributed to the lag of the ensemble temperature response to the change of laser power.

In general, the background (local) temperature rise is attributed to the light absorption in the suspension (nanojet). For example, if the cell has a weak absorption at the working wavelength, the background temperature at two states is close to each other. Therefore, the right-hand side of Eq. (5) would be close to zero with inhibited hysteresis. When the state changes from low power (*n*) to high power (*m*) with infinitely small separation (Figure 2(a)), the system first experiences local heating (LH, inside the nanojet), while the global heating (GH) of the environment comes slower than the local heating (Figure 2(b)). Therefore, the hysteretic response of the backaction force comes from the response difference between the local and global temperature rise. Contrarily, the local cooling (LC) is faster than the global cooling (GC) in the power unloading stage, as illustrated in the temperature diagram (Figure 2(b)).

We adopted a custom-built MLL to study the reversable force under power loading/unloading. The detailed description of the MLL can be found in our previous reports and a brief description in materials and methods [43, 45]. The MLL provides a platform to study interesting nonlinear dynamics, such as the soliton molecule, and optical rogue waves [46, 47], and the major advantage to use the MLL is that with chirped pulse amplification, the peak power can go several orders of magnitude higher than that the continuous wave laser can reach. Moreover, the laser system may have intrinsic hysteresis owing to the thermal effect [48]. We varied the total power through the power amplifying unit to bypass the hysteresis over power adjustment, and the hysteresis elimination has been corroborated by the power measurement in both ascending and descending orders with almost overlapping power readings at the same laser powers. Figure 2(c) illustrates the schematics of the experimental setup for backaction force measurement. We adopted video microscopy illuminated by an LED to record the bright-field image sequence of the microspheres (Figure 2(c)). The detection arm is orthogonal to the laser beam inside the suspension, akin to the light-sheet fluorescence microscopy [49], but with separate visible light from an LED (M530L3, Thorlabs) on the opposite side of the microscope for illumination. The dielectric polystyrene microspheres (5 μm in diameter) were suspended in deionized (DI) water (Millipore, 18.5 MΩ). The microsphere suspension was vigorously shaken and sonicated to ensure the uniformity of dispersion without aggregate. The particle concentration was adjusted to make sure that the particles are neither crowded nor sparse, typically, 40 μL of stock microsphere is suspended into 3 mL pure water for the experiment. The sample cuvette was directly exposed to the collimated light (setup in Figure 2(c)). We performed the PNJ mediated backaction experiment with 5 μm polystyrene microsphere and evaluated the backaction force. The sample video was analyzed using particle tracking algorithm to determine the motion trajectories and average speed over all the particles [50].

In equilibrium, the hydrodynamic Stokes force balances the PNJ-mediated backaction force. Therefore, the back-action force is evaluated through the fluidic drag force, i.e., *F* = 6*πηrv*, with *η* the viscosity of fluid, *r* and *v* are the radius and speed of the microsphere respectively. Note that the viscosity decreases as the ensemble temperature *T*_0_ increases when the incident power is ramped up. Specifically, the viscosity depends on the ensemble temperature, thus the integral of the incident power with respect to time, i.e., the viscosity fluctuates with the accumulation of the absorbed incident laser. We want to minimize the viscosity fluctuation during power adjustment, and thus to simplify the drag force calculation. To do so, we implemented an open cuvette that allows the heat to dissipate to and exchange with the environment, and minimized the measurement time during a single hysteresis cycle such that the total heat absorption will be minimum. As the magnitude of backaction force increases monotonically with laser power, counterintuitively, we found that despite the laser power is the same; the magnitude of backaction force on dielectric particles varies according to the direction of the power change (Figure 2(d)). In particular, the force decreases with a separate trace during power unloading in contrast to power loading. Such distinct force path during power loading/unloading suggests hysteretic response of backaction force in the dielectric particle suspension. This observation well corroborates the theoretical analysis on the hysteresis behavior of the backaction force on the dielectric particles. More precise measurement of the backaction force is also possible to correct for the drag coefficient by recording the ensemble temperature with a thermoresistor in real time, however, this would not change the physical picture of our observation on the force hysteresis, which is corroborated by the speed difference at the same power level but opposite power change direction. Interestingly, this hysteretic power discrepancy originates from the history-dependent particle response.

Next, we explore the force response of biological cells since the cells are the fundamental units in biological tissue. Microscopic particles in the suspension experience fierce collision from the surrounding molecules and perform Brownian motion. To this end, we evaluated human breast cancer line MDA-MB231 (ATCC^®^, HTB-26TM, RRID: CVCL 0062). The cancer cells were cultured in T75 flasks filled with 10 mL of the culture medium (RPMI-1640 with 10% fetal bovine serum and 1% antibiotic-antimycotic) in an incubator with 5% CO_2_ under 37 °C. The cell concentration was adjusted with filtered PBS buffer to be ∼10^6^ cells/mL, and loaded into the glass cuvette for the optical force experiment. The concentration would be further fine adjusted to ensure a reasonable density under the sideview microscope. In the presence of the MLL, the cancer cells are all attracted to the laser source. The maximum strength of force provides a capacity to establish the backaction. Figure 2(e) and (f) demonstrate the hysteresis response of the backaction force on the cancer cells in native and inanimate states evaluated under the illumination of the amplified all-fiber MLL. The magnitude of force is greater in the power loading stage than that in the unloading stage. This distinct force difference suggests a counter-clockwise hysteresis loop (Figure 2(e)). The hysteretic force response implies that the backaction force is not only determined by the average laser power, but also closely related to the previous status that the particle suspension was experiencing. We also replace the standard PBS buffer with and immerse the cells in 70% ethanol solution overnight to dehydrate and fix the cells. Interestingly, the inanimate cancer cell also suggests hysteresis (Figure 2(f)). The force-power curve suggests a counter-clockwise loop for both the live and fixed MB231 cells. The morphology of the MDA-MB231 cells was inspected using scanning electron microscope (Hitachi S.-4800). The cancer cells were immersed in 2.5% glutaraldehyde (GTA) in cacodylate buffer (0.1 M) overnight, and were transferred on the membrane with pore size of 0.8 μm. Ethanol suspensions with a concentration gradient were filtered through the membrane in ascending order followed by a critical point dry (see methods for details). The SEM images suggest that a plethora of trans-membrane proteins with feature size of ∼50 nm are active on the cancer cell membrane (inset in Figure 2(e)). Such feature provides Rayleigh scatterer during light–matter interaction. Once the cancer cells are fixed with 70% ethanol, the transmembrane protein adheres on the membrane and the cell surface becomes smoother and spherical (Figure 2(f)). Since the fixed cancer cells display smoother surface (insets in Figure 2(e) and (f)), and the inanimate MDA-MB231 cells show similar behavior on the backaction force with the live cell, the state of the transmembrane protein is insignificant to create the force preferentially owing to the smaller characteristic size in contrast to the wavelength.

Inhomogeneous cell concentration results in variant number of cells in the video of the same length and insufficient number of statistics causes deviation, and all the power loading/unloading experiments were repeated for more than three times with consistent responses. Our observation on the history dependent backaction force may shed insight on designing photonic device with biological material. The history dependence is the consequence of the erratic and random time evolution characteristic of the hydrodynamic system. In contrast to the deterministic system, including the thin film, nanoparticle aggregates, and the semiconductor lasers [48, 51], the colloidal hydrodynamic suspension provides a novel platform to investigate the nonlinear phenomenon. The parallel manipulation ability allows the creation of photonic devices using massive biological cells, and most importantly, the parallel manipulation has no restriction in terms of the spatial region.

### 2.2 Backaction force with distinguishable spatiotemporal lasers

**Figure 3:**
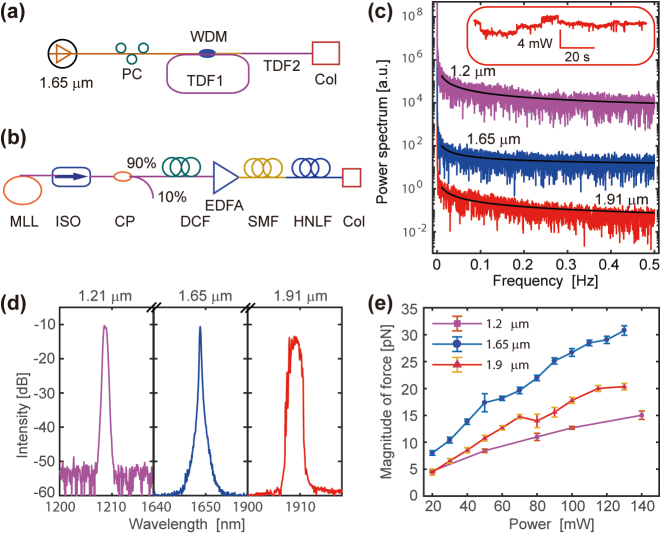
Schematic of the multi-line backaction force. (a) The layout for a custom-built fiber laser operating at a central wavelength of 1.91 μm. PC, polarization controller; WDM, wavelength division multiplexer; TDF, thulium-doped fiber; Col, collimator. (b) Schematic layout for the supercontinuum generation. ISO, isolator; CP, coupler; DCF, dispersion compensating fiber; EDFA, erbium doped fiber amplifier; SMF, single mode fiber; HNLF, high nonlinear fiber. (c) Power spectrum of the 2-stage TDF fiber laser (1.91 μm wavelength, red curve) suggests pronounced noise at extremely low frequency range. The power spectrum approximates an *f* ^*α*^ noise. Inset shows the power fluctuation with respect to time for the TDF laser. (d) The optical spectra of the three laser lines operating at continuous wave used for the backaction force experiment. (e) The magnitude of optical force on 10 μm diameter polystyrene microsphere increases monotonically with the laser power for the respective laser lines.

The PNJ can evaporate the liquid molecules with ultrashort femtosecond laser pulses to generate the air bubble which pushes the particle backwards [44]. Our observation suggests that the PNJ mediated backaction would also take place with a custom-built all-fiber MLL, for which the peak power is much weaker than that for the ultrafast femtosecond pulses [43]. It would always be great to reduce both the average power and the peak power of the manipulation beam such that the system can work safely with biological cells. Naturally, people may ask whether the PNJ-mediated backaction would take place with continuous wave laser. Here, we explored the use of a near infrared II laser with distinguishable spatiotemporal profile to excite the backaction with PNJ [52]. We first built an all-fiber resonator in the mid-infrared region using thulium doped fiber (TDF, 50 cm length), and amplified the laser using an external TDF (80 cm length) outside the cavity to absorb the residual pump (Figure 3(a)). The TDF fiber laser was pumped by a Raman fiber laser (see methods for details). The second laser we built is an all-fiber supercontinuum laser source with self-phase modulation in high-nonlinear fiber (layout in Figure 3(b), and description in Materials and Methods). In brief, the supercontinuum laser starts from the MLL [43, 45] with central wavelength of 1.55 μm, and repetition rate of 44 MHz. The supercontinuum beam would further be achieved by passing the amplified pulses through a piece of SPINE-HNLF (Figure 3(b)). The produced supercontinuum beam preserves the same repetition rate as corroborated in the pulse sequence monitored after the SPINE-HNLF. The 20 dB bandwidth for the supercontinuum is over 400 nm, with a nearly flat spectrum between 1700 and 1900 nm.

As comparison, we also employed two commercial fiber lasers with separate lines to perform the PNJ-mediated backaction. The first laser is an ytterbium fiber laser with a Raman wavelength shifter (PYL-1-1209, IPG Photonics) operating continuously at central wavelength of 1.21 μm with random polarization. The second is a commercial Raman fiber laser (KEOsys.) with a central wavelength of 1.65 μm. Since the two commercial lasers and the TDF fiber laser are all operating in the continuous mode, we compare the power stability of all these laser lines. The spectrum density of the output power for the two-stage TDF fiber laser suggests an *f* ^*α*^ noise with 0.5 < *α* < 1 and 2 h power stability of ∼0.8% (Figure 3(c)). The pronounced low-frequency noise (*f* < 0.02 Hz) non-overlapping with the detection range for Brownian motion (∼0.2–100 Hz), contributes insignificantly to the detection noise. Figure 3(d) shows the optical spectra for all the three lasers running under continuous wave. The commercial Raman lasers (1.21 and 1.65 μm) suggest ultranarrow bandwidth and high degree of temporal coherence, while the custom-built gain-switched TDF fiber laser provides broadband spectrum and poor coherence. Such low coherence is attributed to the two-stage laser pump absorption and the continuous pump (Figure 3(a)).

Next, we performed the backaction force experiment with either the commercial laser lines or the custom-built lasers. All the laser lines, operating under continuous wave mode, were delivered to free space via a fiber collimator. The collimated beam directly entered the cuvette containing the dielectric microparticle suspension (optical path length 10 mm). Interestingly, we found that the laser with both low and high coherence can be used to pull the dielectric spheres towards the light source. The custom-built all-fiber laser centered at 1.91 μm with a much broader spectrum as compared with the commercial Raman lasers (Figure 3(d)) can also pull the microspheres towards the light source. This PNJ mediated force not only takes place with pulsed laser, but also appears with laser under continuous wave operation. The laser lines with apparent particle backaction are within the near-/mid-infrared region, for which the water absorption is much greater than the visible wavelength. Both the Raman lasers with wavelength 1.21 and 1.65 μm provide backaction force on the polystyrene microsphere. The magnitude of force with 1.65 μm laser is stronger than the laser with 1.21 μm wavelength at the same injected laser power. To mention that the light absorption in the dielectric suspension is strong at these wavelengths; such backaction was not observed with light in the visible range operating in continuous wave.

Moreover, the magnitude of the force shows different behavior for each wavelength. In brief, at the same laser power, the laser with 1.65 μm wavelength provides stronger force, while the 1.21 μm laser provides smaller magnitude of force. The laser of 1.91 μm wavelength produces intermediate force strength (Figure 3(e)). The alteration of laser wavelength suggests distinguishable magnitude of backaction force, which is the consequence of the interplay between light absorption and thermo conductivity. As the thermo-conductivity is independent on the wavelength, the change of backaction force attributes to the variable absorption in the solvent at respective wavelength. The water absorption spectrum suggests that *α*_1.2_ < *α*_1.65_ < *α*_1.9_ in the near infrared region [53]. For all the power levels under test, the magnitude of backaction force follows *F*_1.65_ > *F*_1.9_ > *F*_1.2_, and such experimental observation is contradictory with our previous explanation on the absorption dependent backaction force. We further inspect that the transmission of the polystyrene sphere at 1.91 μm wavelength (∼70%) is much smaller than that at 1.65 μm (∼90%). Thus, our observation of the difference on wavelength dependent backaction force is well explained by the combined effect of light transmission of the dielectric particle and the light absorption inside the nanojet (Figure 3(e)). Moreover, we also found that the supercontinuum source with distinguishable spatiotemporal profile could also mediate backaction force on the dielectric particle.

### 2.3 Force balance with counter-propagating beams

**Figure 4:**
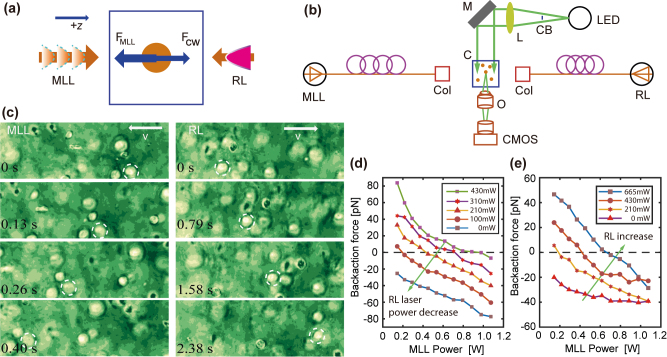
Force balance on cancer cells under counter-propagating beams. (a) Demonstration of the backaction force on the microsphere exerted by the counter-propagating beams. (b) Experimental setup with MLL and Raman lasers performing backaction force from opposite directions. The particle motion was observed by an orthogonal microscope under bright field. MLL, mode-locked laser; C, cuvette; RL, Raman laser; M, mirror; L, lens; CB, central block; O, objective; LED, light emitting diode. (c) Motion snapshots of live MDA-MB231 cancer cells under paired beams with MLL laser (left) or the Raman laser (right, wavelength of 1.65 μm) dominant suggest opposite motion directions. The average speed for the cancer cell marked in dashed circle moving towards left (right) is ∼482.2 μm/s (∼59.5 μm/s). (d) The optical force for the polystyrene microsphere of 15 μm diameter as function of the MLL laser power with the Raman laser kept fixed at 0, 100, 210, 310, and 430 mW respectively. The particle motion direction reverses once the power from the Raman laser is strong enough to provide greater backaction force towards the right. (e) The large-scale optical manipulation of the MDA-MB231 cells using the counter-propagating beams suggests the balance of the cancer cell under proper combination of the power of the two beams.

The backaction force moves the particle against the light photon flux direction. In order to slow down the particle motion and provide stable manipulation with the PNJ, we demonstrate that two lasers with distinct spectral and temporal profiles can produce balanced backaction force. The experimental configuration was designed with two counter-propagating beams shining the dielectric suspension from opposite directions (Figure 4(a)). On the left hand side, we exerted the backaction force with an amplified custom-built MLL fiber laser (center wavelength 1576 nm, repetition rate 44.5 MHz) [43], while the particles were pulled to the right hand side by a Raman laser (RL, Keopsys) operating at continuous wave with center wavelength of 1.65 μm. The motivation for the combination is to effectively minimize the coherent interaction between the beams. A complete experimental setup is shown in Figure 4(b). A relay lens (*f* = 200 mm) collimates the LED light onto the sample cuvette. A center block (CB) was introduced to partially block the illumination light with small *k* vector (Figure 4(b)) to reduce the direct scattered light and to increase the contrast of cell images. The detection microscope consists of an objective lens (Olympus, 20×, NA = 0.45), a CMOS camera (DC1645c-HQ, Thorlabs), and a 25 mm TV lens (Electrophysics TV lens) (Figure 4(b)). The images were acquired at a speed of 20–50 fps depending on the field of view, and saved on the computer for offline particle tracking and analysis [50].

Both laser lines were flexibly delivered through respective fibers. Here, we elect the propagation direction of the MLL as the “+z” direction. The backaction force originating from the MLL points to the left side (“−z”). The backaction force from the continuous RL laser points to the “+z” direction (opposed to the photon flux from the right laser source) (Figure 4(b)). By judicious choice of the power combination for both beams, the backaction force on microsphere could eventually balance each other. The Brownian motion becomes more augmented as the power for the counter-propagating beams increases. In the absence of the MLL, the particle speed increases monotonically with the power of the Raman laser (Figure 2(e)). The ability to attract particle has been verified with polymer spheres of various sizes (5, 10, 15 μm in diameter). The microparticles all swam towards the left in response to the ML. The particle motion speed increases as the laser power ramps up. In contrast, the particle motion direction reverses in the presence of the solely Raman laser. The average speed of the particle under the illumination of the Raman laser also increases with the laser power (Figure 3(e) for 10 μm polystyrene microsphere).

Here, we select the breast cancer cells (MDA-MB231) to perform the experiment, and adopt the MLL to mediate the backaction force on the biological cancer cells. The pulling of cancer cells suggests that the cells move in response to the biophotonic nanojet (BioPNJ), although the refractive index contrast of the biological cells with respect to the background medium is much smaller than that of the standard polymer sphere (most biological material has a relative refractive index of 1.02–1.05) [54]. The pulling of the cancer cells by counter-propagating beam has been corroborated experimentally (Supplemental Video S1 and S2). Figure 4(c) shows the snapshots in the video of the MDA-MB 231 cancer cells that move towards left (right) in response to the MLL (RL) laser. The white arrows on each image indicate the motion direction (opposite to photon flux of the dominant beam). The video snapshots imply that the cells can be manipulated by counter-propagating beams with reversed direction. Here, as demonstration, we kept the power of the right RL fixed at 211 mW. When the left MLL power is dominant, i.e., at 616 mW in Supplementary Video S1 and the left column in Figure 4(c), all cancer cells move towards left. The average speed for the cell marked in dashed circle is 482.2 μm/s. Once the power of the left laser reduces to 143 mW, the right RL is dominant, as consequence, all the cancer cells move towards right with an average speed for the cancer cell marked in dashed circle of ∼59.5 μm/s (Supplementary Video S2, and right column of Figure 4(c)). Although we marked a single cancer cell with dashed circle, all the cells in the field of view move in parallel towards the light source of the strong beam. Therefore, the PNJ mediated optical backaction provides parallel manipulation, as long as all the particles exposed in the collimated light field. In the absence of the Raman laser (right), the magnitude of backaction force increases with the power of the MLL (polymer microsphere in Figure 4(d), and MDA-MB231 cell in Figure 4(e)).

**Supplementary Video S1:** 

**Supplementary Video S2:** 

Tug-of-war on biomolecule and individual bacterium has been demonstrated using a dual-trap optical tweezers [55–57] and the holographic elongated dual-trap tweezers [58]. The existing technique is optimized for single particle (protein, nucleic acid, or bacterium) manipulation, and requires sophisticated control (high-precision stage or spatial light modulator) [56, 58]. However, in cancer cell studies, collective particle manipulation is required to achieve large-scale and high-throughput biopsy and classification. The PNJ assisted backaction force with counter-propagating beam provides force balance on particles in large-scale.

We next understand the competing effect of the two beams by fixing the power of the Raman beam and altering the power of the MLL. When the continuous Raman laser shined the suspension from the right-hand side, the particles all moved towards the Raman laser source. In presence of the Raman laser with fixed power, the curve shifts upward depending on the power of the Raman laser. The shifted curve intersects the horizontal abacas with a balanced power of the MLL (dashed line in Figure 4(d) and (e)). At these balanced points, lasers from the counter-propagating beams produce equal magnitude of backaction force. Therefore, the total effective force from two beams would be zero. In contrast to the forward radiation pressure force that originates from the counter-propagating beams and balances each other, the backaction force mediated by the PNJ with two counter-propagating beams can also balance each other by precise choice of the power ratio (schematics in Figure 4(a)). In fact, the powers for the two beams to create balanced backaction force are non-identical owing to the discrepancy in the light absorption in the solvent and the focusing ability of the microsphere at respective wavelengths. However, the balance point can be found by keeping the power for one of the beams (e.g., Raman laser) fixed, while altering the power of the other beam (e.g., MLL laser) gradually and checking the average motion speed of the particles until the speed gently decreased to zero. At this balance position, the particles only experience Brownian motion due to the collision from liquid molecules. The magnitude of Brownian motion augments as the balance power increases since the overall ensemble temperature ramps up at high injected power.

Consequently, as the power of the Raman laser increases, the balance point shifts to the right, suggesting that a greater power of the MLL laser is required to balance the Raman laser. This competing force from the two beams has been observed not only with polymer microspheres (Figure 4(d)), but also with the biological cancer cells, i.e., MDA-MB231 cells in Figure 4(e). Potential application would be the multi-dimensional manipulation of the cells for cell surgery and cell opto-mechanics. The speed response of the MDA-MB231 cells shows weak saturation owing to the photothermal lens effect [43]. The speed even slightly overlaps at high power levels. This takes place stochastically as the standard polymer microsphere and cancer cells produce more promising trends. Another possible reason is that the accuracy of the speed depends on the particle density. As the particle sediments over the experimental period, the particle density in each video may vary (basically decreases with time). At the beginning, when the particle density is high, the average speed has been averaged over more numbers of particles. While the time elapses, the particle density decreases significantly due to sedimentation, the speed measurement becomes less accurate owing to the reduction of the number of particles.

### 2.4 Reversible backaction originates from hysteresis under counter-propagating beams

**Figure 5:**
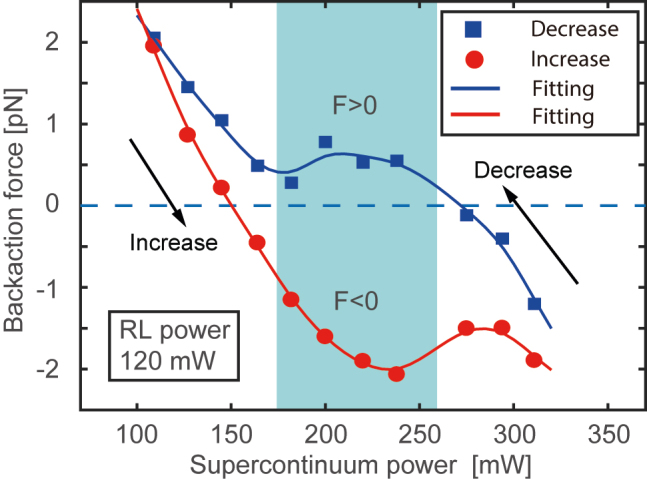
Reversible backaction force on the polystyrene microsphere of 10 μm diameter controlled by hysteresis. The backaction force changes with the direction of power change of the supercontinuum source, while the counter-propagating Raman laser with wavelength of 1.65 μm was fixed at a power of 120 mW. Blue squares and red circles demonstrate the experimental measurement when the power of the supercontinuum beam reduces and increases respectively. The blue and red solid lines show the fitting.

It is recently of great interest to use the super-luminescent or even white light for optomechanics and optical binding studies [59, 60]. Our demonstration on the multiple laser lines in the mid-infrared region suggests the possibility to manipulate the cell motion using the supercontinuum laser source that spans in this spectral region with strong water absorption. As the hysteresis is associated with the distinguishable response to the temperature between the nanojet and the ensemble, such phenomenon is not related to the spatiotemporal profile of the pulse sequence. This has been corroborated by the observation of the hysteresis on the backaction force induced by the laser beam with supercontinuum spectrum. The experimental setup is similar to the counter-propagating beam setup (Figure 4(b)). Specifically, we replaced the MLL with the developed supercontinuum laser source, and kept the RL (cw, center wavelength of 1.65 μm) on the right-hand side. The RL fixed at a certain power provides a constant force on the particle. In presence of the RL (from right side in Figure 4(a)), the backaction force created by the supercontinuum source also suggests hysteresis. Figure 5 shows the backaction force on the polystyrene microsphere with 10 μm diameter. The blue squares and red circles represent the measurement for backaction forces produced by the supercontinuum beam in presence of the right RL of 120 mW power with decreasing and increasing power respectively. For the supercontinuum beam power between 170 and 260 mW (shaded area), the backaction force is reversibly determined by the power change direction. Therefore, the reversible force would be manipulated by the hysteresis of backaction force on the dielectric particle. In contrast to the mode-locked laser, the supercontinuum laser was configured at a lower power with considerably good stability, the precision of the backaction force could be better than sub-1 pN. This allows the precision measurement of the backaction force on the dielectric particle.

Moreover, the supercontinuum source has smaller power range as compared to the MLL laser used in the single beam hysteresis experiment in Figure 2. Such lower power range offers much smaller increase in global temperature *T*_0_, thus very limited viscosity fluctuation. This further corroborates the presence of hysteresis on the backaction force mediated by the PNJ. Finally, single beam optical tweezers only trap particles near the narrow diffraction-limited focus. In contradistinction, the PNJ innately comes with and moves along with the microsphere (Figure 1(b)). As long as the particle is shined by light, the particle will be passively “self-powered” and respond to the backaction force. The backaction force can be balanced through precise control over the power combination and be reversed by the hysteresis. Therefore, the near-/mid-infrared laser provides large-scale manipulation and reversible control of dielectric particles and biological cells. In combination with the distinct backaction force from different laser lines, the supercontinuum spectrum provides opportunities to augment the backaction force on the dielectric particles with a broad size range from microns to milli-meters.

## 3 Conclusions

In conclusion, the reversible backaction force on dielectric particle and cancer cells could be achieved by the force hysteresis during the loading/unloading of the laser power. The hysteresis is attributed to the difference in temperature response between the nanojet and the ensemble, and may potentially be applied for optical memory, data storage, optical bistability using the soft matter suspension of dielectric microparticles [14, 61]. Such behavior shed new insights on novel mesoscale devices using soft matter that are reconfigurable by light. In general, the light–matter interaction with grafted particle or particle decorated with structured plasmonic layers, in addition to the spatially and temporally structured light, is also interesting to explore [62]. Moreover, we observed that the continuous wave laser can also mediate the backaction force as long as the liquid in the PNJ absorbs the light photons. Such backaction force can be balanced by using two counter propagating beams even with different spectral and temporal profiles. The backaction force from counter-propagating beams with dual color can also balance the motion of biological cells with subtle refractive index difference with the background medium.

The microsphere is propelled by local energy conversion inside nanojet and moves autonomously as long as the sphere refractive index is greater than that of the background medium. Biological settings provide abundant dielectric particles, i.e., cells and organelles, in the range from nanometers to hundreds of microns. Using light to remotely control the cell motion and to detect the motion behavior is of utmost importance in biomedical applications. One promising application is the identification of cancer cells which relies on a plethora of biological hallmarks to delineate the key features. The backaction force enabled by the PNJ also allows the directional motion of biological cells for targeted cell surgery.

## 4 Materials and methods

### 4.1 All fiber MLL

In brief, the all fiber resonator consists of an erbium doped fiber (EDF) with low doping (3.6 m, Levent-SFD), a piece of single-mode fiber (SMF, 0.5 m, Corning, HI1060), and a standard SMF (0.5 m, Corning, SMF 28e). With a 976 nm single-mode laser diode (LD) as the pump, the all fiber MLL supplies pulses with center wavelength of 1.55 μm, and repetition rate of 44.5 MHz [45]. Nearly half of the output was extracted from the fiber cavity through a single optical integrated module. The laser output passes through a fiber isolator to eliminate back reflection and 9:1 coupler to split the energy for amplification and sampling. 90% of the output was then amplified by a commercial L-band erbium-doped fiber amplifier with final central wavelength of 1576 nm (EDFA, IPG photonics, bandwidth 1570–1605 nm) [43]. The pulse train was coupled to free space for the optical manipulation via an optical fiber collimator (Figure 2(c)).

### 4.2 Continuous wave all-fiber laser sources

We built a continuous wave all-fiber resonator with a piece of thulium doped fiber (TDF, 50 cm length) as the gain medium, and further amplified the laser using another piece of external TDF (80 cm length) outside the cavity owing to the absorption of the residual pump (Figure 3(a)). The custom-made TDF fiber laser was pumped by a Raman fiber laser (KEOsys., wavelength 1.65 μm). The pump laser was coupled into the fiber cavity through a wavelength division multiplexer (WDM), while a piece of 50 cm TDF inside the fiber cavity acts as the gain medium. The laser was coupled out of the fiber cavity through the same WDM, and collimated through a fiber collimator. The collimated beam shines the dielectric microparticle suspension in the cuvette (Figure 2(c)). The TDF laser has an output central wavelength of 1.91 μm (see the laser spectrum in Figure 3(d)). Since the continuous wave laser would be considered as gain-switched TDF laser with stochastic laser switching [63, 64] and the power stability would not be comparable as that of the Raman lasers, we evaluate the power stability by recording the output power fluctuation, which suggests a 2 h stability of ∼0.8%. The noise spectrum (Figure 3(c)) suggests a 1/*f* ^*α*^ noise with 0.5 < *α* < 1.

### 4.3 Supercontinuum laser source

The supercontinuum laser starts from the MLL [43, 45], which supplies pulse sequence with central wavelength of 1.55 μm, and repetition rate of 44 MHz [45]. 50% of the output was coupled out from the fiber cavity through a single-mode optical integrated module (OIM). The full-width-at-half-maximum (FWHM) of laser pulse was about 1.6 ps measured with an autocorrelator (Femtochrome, FR-103 MN). The all-fiber MLL pulse sequence passes through a fiber isolator to inhibit back reflection and 9:1 coupler to split the energy for amplification and sampling. 90% of the output energy was further stretched by a piece of dispersion compensating fiber (DCF, length 1.46 km, 0.54 dB loss, −166 ps/nm dispersion), amplified by a commercial EDFA (IPG photonics, bandwidth 1570–1605 nm), and compressed by another piece of SMF (11.355 km, dispersion 178.7 ps/nm). The pulse train was coupled to free space for the optical manipulation experiment via fiber collimator. The optical spectrum was evaluated by sending the pulsed beam into an optical spectrum analyzer (OSA, Yokogawa AQ6375). Optical spectrum before amplification suggests a 3 dB bandwidth of 53 nm. Only the strong portion of the original spectrum was amplified, producing pulsed beam with a central wavelength of 1576 nm, and a 3 dB spectral bandwidth of 18 nm and variable output from 0.3 to 2 W. The amplified temporal pulse train was detected by a 10 GHz high speed photodetector (HiPD, HP 11982A), and was digitalized by a real time oscilloscope (Lecroy SDA 820Zi-B). The pulses have a repetition period of 22.5 ns corresponding to a repetition rate of 44.5 MHz.

### 4.4 Cancer cell culture and characterization

The MDA-MB231 cancer cells were cultured in T75 flasks with 10 mL of culture medium (RPMI-1640 with 10% fetal bovine serum and 1% antibiotic-antimycotic) in an incubator with 5% CO2 under 37 °C. The cell suspension in growth medium were washed three times in filtered PBS buffer and adjusted to a cell concentration of ∼10^6^ cells/mL. The cell suspension was loaded into the glass cuvette for the optical pulling experiment. The concentration is also adjusted to ensure a reasonable density under the sideview microscope. We have inspected the morphology of the cells using scanning electron microscope (Hitachi S.-4800). The cancer cells were immersed in 2.5% glutaraldehyde (GTA) in cacodylate buffer (0.1 M) overnight at 4 °C. The cell suspension was diluted with ∼3 mL 30% ethanol and transferred on the membrane with pore size of 0.8 μm. Ethanol suspensions with a concentration gradient of (30, 50, 70, 90, 100%) were gradually filtered through the membrane to replace water in the cells. A critical point dry to remove the ethanol followed by sputter coating of the conductive layer was performed before the SEM image. The cancer cell in native environment has a plethora of trans-membrane protein, for which the feature size is on the order of 50 nm (inset in Figure 2(e)), however, the cell surface becomes smoother when the cells are fixed with GTA (inset in Figure 2(f)).
